# Standard versus delayed initiation of S-1 adjuvant chemotherapy after surgery for pancreatic cancer: a secondary analysis of a nationwide cohort by the Japan Pancreas Society

**DOI:** 10.1007/s00535-023-01988-7

**Published:** 2023-06-17

**Authors:** Yoshito Tomimaru, Hidetoshi Eguchi, Yoshimitsu Shimomura, Tetsuhisa Kitamura, Yosuke Inoue, Yuichi Nagakawa, Akihiro Ohba, Shunsuke Onoe, Michiaki Unno, Daisuke Hashimoto, Shoji Kawakatsu, Tsuyoshi Hayashi, Ryota Higuchi, Hirohisa Kitagawa, Kenichiro Uemura, Yasutoshi Kimura, Sohei Satoi, Yoshifumi Takeyama, the Committee of Clinical Research, Japan Pancreas Society

**Affiliations:** 1 Department of Gastroenterological Surgery, Graduate School of Medicine, Osaka University, 2-2 Yamadaoka E-2, Suita, Osaka 565-0871 Japan; 2 Division of Environmental Medicine and Population Sciences, Department of Social and Environmental Medicine, Osaka University, Suita, Japan; 3 Department of Hematology, Kobe City Organization Kobe Medical Center General Hospital, Kobe, Japan; 4 Division of Hepatobiliary-Pancreatic Surgery, Cancer Institute Hospital, Tokyo, Japan; 5 Department of Gastrointestinal and Pediatric Surgery, Tokyo Medical University, Tokyo, Japan; 6 Department of Hepatobiliary and Pancreatic Oncology, National Cancer Center Hospital, Tokyo, Japan; 7 Division of Surgical Oncology, Department of Surgery, Nagoya University Graduate School of Medicine, Nagoya, Japan; 8 Department of Surgery, Tohoku University Graduate School of Medicine, Sendai, Japan; 9 Department of Surgery, Kansai Medical University, Hirakata, Japan; 10 Department of Gastroenterological Surgery, Aichi Cancer Center Hospital, Nagoya, Japan; 11 Center for Gastroenterology, Teine-Keijinkai Hospital, Sapporo, Japan; 12 Department of Surgery, Institute of Gastroenterology, Tokyo Women’s Medical University, Tokyo, Japan; 13 Department of Surgery, Kurashiki Central Hospital, Kurashiki, Japan; 14 Department of Surgery, Graduate School of Biomedical and Health Sciences, Hiroshima University, Hiroshima, Japan; 15 Department of Surgery, Surgical Oncology and Science, Sapporo Medical University School of Medicine, Sapporo, Japan; 16 Division of Surgical Oncology, University of Colorado Anschutz Medical Campus, Aurora, CO USA; 17 Department of Surgery, Kindai University, Osaka, Japan

**Keywords:** Pancreatic cancer, Adjuvant chemotherapy, S-1, Initiation, Survival

## Abstract

**Background:**

Based on the Japan Adjuvant Study Group of Pancreatic Cancer-01 results, S-1 adjuvant chemotherapy has been the standard in resected pancreatic ductal adenocarcinoma (PDAC) patients in Japan and elsewhere, initiated within 10 weeks after surgery. To assess the clinical impact of this timing, we conducted a secondary analysis of a nationwide survey by the Japan Pancreas Society.

**Methods:**

A total of 3361 patients were divided into two groups: 2681 (79.8%) initiating the therapy within 10 weeks after surgery (standard) and 680 (20.2%) after 10 weeks (delayed). We compared recurrence-free survival (RFS) and overall survival (OS) using the log-rank test and Cox proportional hazards model with conditional landmark analysis between the groups. Results were verified by adjustment with inverse-probability-of-treatment weighting (IPTW) analysis.

**Results:**

The median timing of S-1 adjuvant chemotherapy initiation was 50 days (interquartile range: 38–66). In the standard group, 5-year RFS and OS rates were 32.3–48.7%, respectively, compared with 25.0–38.7% in the delayed group. Hazard ratios (HRs) and 95% confidence intervals were 0.84 (0.76–0.93) for RFS (*p* < 0.001) and 0.77 (0.69–0.87) for OS (*p* < 0.001). The IPTW analysis yielded 5-year RFS rates of 32.1% and 25.3% in the standard versus delayed group, respectively [*HR* = 0.86 (0.77–0.96), *p* < 0.001] and 5-year OS rates of 48.3% and 39.8%, respectively [*HR* = 0.81 (0.71–0.92), *p* < 0.001].

**Conclusions:**

Initiation of S-1 adjuvant chemotherapy in resected PDAC patients within 10 weeks after surgery may offer survival benefit over later initiation.

**Supplementary Information:**

The online version contains supplementary material available at 10.1007/s00535-023-01988-7.

## Introduction

Pancreatic ductal adenocarcinoma (PDAC) is one of the main causes of cancer-associated mortality worldwide, and the prognosis is dismal, with a 5-year overall survival (OS) rate of < 10% [1]. Resection contributes to the chance of cure, but survival rates even after the resection remain extremely low [2–5]. To improve the prognosis, some groups have evaluated clinically effective adjuvant chemotherapy in resected PDAC cases [6–10]. S-1 is a pro-drug of 5-fluorouracil (5-FU), consisting of tegafur, gimeracil (CDHP; an inhibitor of dihydropyrimidine dehydrogenase catabolizing 5-FU), and oteracil (an inhibitor of phosphorylation of 5-FU) [9, 11]. The Japan Adjuvant Study Group of Pancreatic Cancer (JASPAC)-01 findings demonstrated that adjuvant chemotherapy with S-1 offered survival benefits compared with gemcitabine in resected PDAC patients [12]. Since that study, S-1 adjuvant chemotherapy has been considered the standard for treating this patient population in Japan and some other Asian countries.

Beyond the strategy employed in the JASPAC-01 study, however, evidence is limited regarding the best protocol for S-1 adjuvant chemotherapy administration in these patients. An example is the duration of administration of S-1 adjuvant therapy, with 6 months considered the standard based on the JASPAC-01 protocol. We recently reviewed real-world data from a large cohort of PDAC patients in Japan to obtain hints for optimizing the duration. Our review indicated that extending the period of S-1 adjuvant chemotherapy beyond 6 months offered no significant additional survival benefit [13]. However, other factors remain to be optimized, including timing of S-1 initiation. In JASPAC-01, S-1 adjuvant chemotherapy was begun within 10 weeks after surgery, and patients with later starts were no longer eligible to participate [12]. As a result, the standard initiation timing is now simply considered to be within 10 weeks after surgery in patients with resected PDAC. Strictly speaking, however, the benefit of S-1 adjuvant chemotherapy shown in that study is applicable only to patients treated in that initiation time frame, and whether equivalent benefit is possible with post-surgery initiation after 10 weeks is unknown. To date, several studies have addressed the timing of adjuvant chemotherapy, but with some weaknesses in study designs [14–17]. Thus, no solid evidence is available regarding the optimal initiation timing for S-1 chemotherapy in terms of postoperative survival in PDAC patients.

Here, we used analysis of real-world patient data to investigate the survival impact of initiating S-1 adjuvant chemotherapy within 10 weeks after surgery compared with starting the therapy after 10 weeks. This study was performed as a secondary analysis of a nationwide survey by the Japan Pancreas Society of a large cohort patients who underwent surgery for PDAC followed by S-1 adjuvant chemotherapy in board-certified institutions.

## Methods

### Study design

**Fig. 1:**
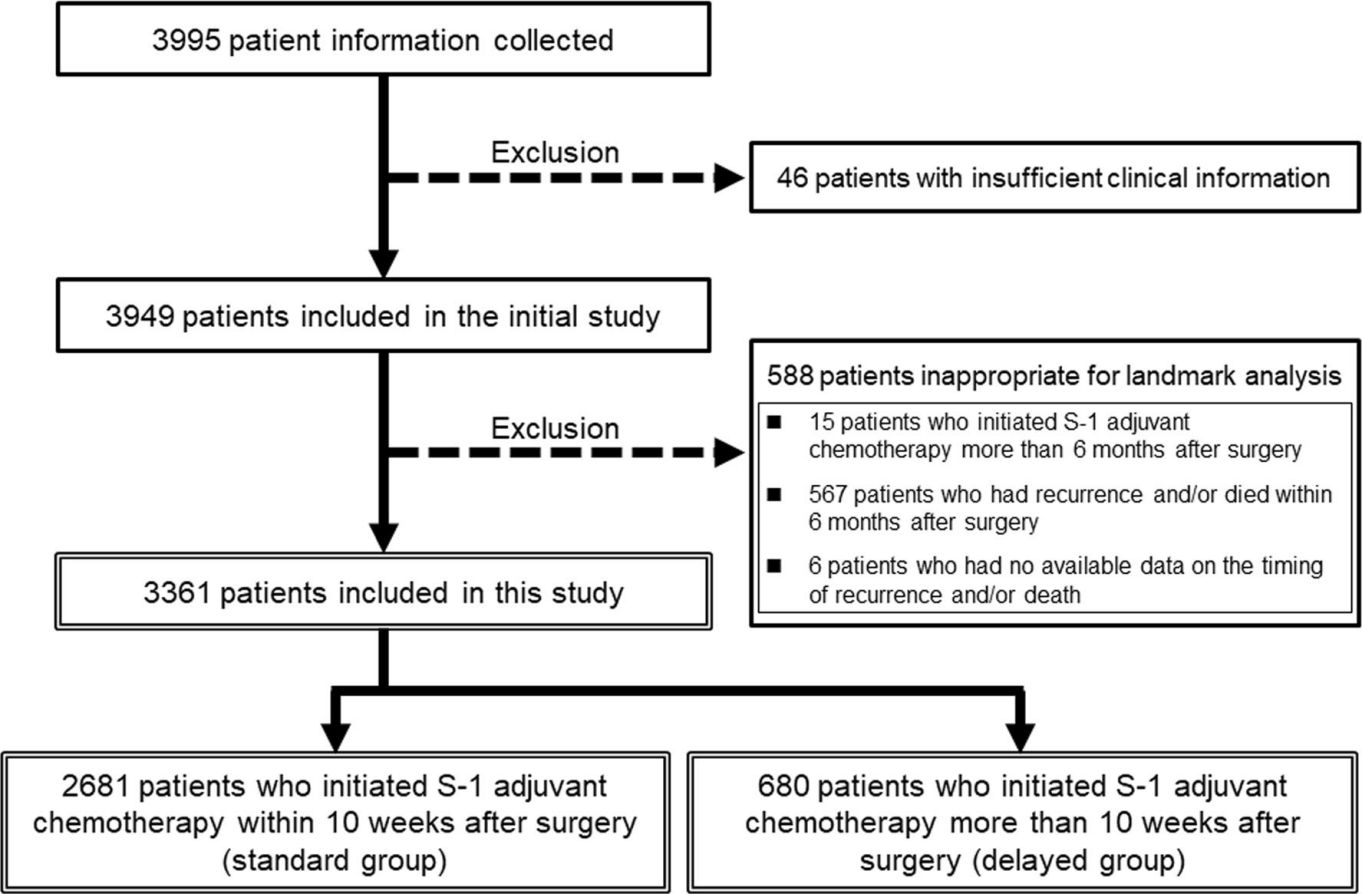
Flow diagram of patient inclusion. Among 3949 patients, 588 were excluded in landmark analysis. The remaining 3361 were divided into two groups: 2681 patients (79.8%) initiated S-1 adjuvant chemotherapy within 10 weeks after the surgery (standard group), and 680 patients (20.2%) did so beyond 10 weeks after the surgery (delayed group). PDAC: pancreatic ductal adenocarcinoma

This study was performed as a secondary analysis of survey data from a project of the Committee of Clinical Research of the Committee for Pancreatic Cancer Registry of the Japan Pancreas Society. Survey data represented a large cohort of patients who underwent surgery for PDAC followed by S-1 adjuvant chemotherapy in board-certified institutions [13]. In the original study, clinical information was collected for 3995 patients who received the treatment from January 2014 to December 2018 at 82 institutions that were board-certified for pancreatology by the Japan Pancreas Society. Because of the inclusion criteria in that study, patients with distant metastasis, R2 resection, or carcinoma in situ were excluded. Of the 3995 patients, 46 were excluded for insufficient clinical information, and the remaining 3949 were included in that study. In the current investigation, we also excluded an additional 15 patients who initiated S-1 adjuvant chemotherapy more than 6 months after surgery, and for a landmark analysis for removing immortal time bias, we excluded 567 patients with recurrence or death within 6 months after surgery and 6 patients who had no available data on the timing of recurrence or death. The remaining 3361 patients were included in this study (Fig. 1). In this cohort, we evaluated preoperative, intraoperative, and postoperative information including OS and recurrence-free survival (RFS) after the surgery. This analysis was approved by the Institutional Review Board (IRB) of Osaka University Hospital together with the initial study (IRB No. 20555) and conformed to the Declaration of Helsinki [18]. Based on the IRB approval, patient consent to participate was obtained using the opt-out method.

### Definitions used in this study

The included patients were divided into two groups based on the timing of initiating S-1 adjuvant chemotherapy: a standard initiation time, defined as within 10 weeks after the surgery (standard group), and a delayed initiation time, defined as starting beyond 10 weeks after the surgery (delayed group). The S-1 adjuvant chemotherapy was administered as previously described [13]. RFS was defined as the time elapsed from the date of surgery to the date of recurrence, and OS was defined as the time elapsed from the date of surgery to the date of death. PDAC staging was based on the UICC TNM classification, and the resectability status of PDAC was defined using the National Comprehensive Cancer Network Guidelines [19, 20]. Eastern Cooperative Oncology Group Performance Status (ECOG PS) was determined as previously described [21], and postoperative complications were defined as any complication during hospitalization rated higher than grade III, using the Clavien–Dindo classification system [22].

### Statistical analysis

Measured characteristics were described using median and interquartile range for continuous variables and numbers for categorical variables. Differences between groups were assessed with the chi-square test, Fisher’s exact test, or the Mann–Whitney U test. We conducted the landmark analysis for removing immortal time bias and set the landmark time at 183 days after surgery [23]. Event rates were estimated with 95% confidence intervals (CIs) using the Kaplan–Meier method for RFS and OS. The impact of standard initiation of S-1 adjuvant chemotherapy compared with delayed initiation was estimated using univariate Cox proportional hazards models for the RFS and OS. The endpoints were described as the hazard ratios (HRs) and 95% CIs. In addition, we established a weighted Kaplan–Meier method and Cox proportional hazards regression model with inverse-probability-of-treatment weighting (IPTW) to reduce bias related to patient background and potential confounding in the direct comparisons [24, 25].

We first calculated the propensity score using a multivariable logistic regression analysis. Factors listed in Table 1 were selected as covariates that could clinically influence the selection of the conditioning regimen. The weights for the delayed group were the inverse of the propensity score, and the weights for the standard group were the inverse of (1—propensity score). To measure the covariate balance, we checked the standardized mean differences (SMDs) before and after matching. SMD values < 0.1 (10%) were considered to indicate a negligible imbalance between the two groups. We plotted the weighted Kaplan–Meier curves of RFS and OS for both groups. The impact of standard versus delayed initiation was estimated using the univariate Cox proportional hazards models for RFS and OS and described with adjusted HRs and 95% CIs. We then performed a subgroup analysis using the Cox proportional hazards model to examine the effect of initiation time in each subgroup and the influence on RFS and OS of interactions between initiation time and the factors listed in Table 1. We additionally compared RFS and OS among multiple groups stratified by initiation time of the adjuvant chemotherapy: ≤ 6 weeks, 6–10 weeks, 10–14 weeks, and > 14 weeks. We estimated event rate using the Kaplan–Meier method for RFS and OS and compared the four groups using the log-rank test. We also performed sensitivity analysis because the optimal cut-off level for initiation time of adjuvant chemotherapy was unknown [14–17]. In the sensitivity analysis, we adopted two other cut-off values for the initiation time of S-1 adjuvant chemotherapy to define two other standard and delayed groups (8 weeks and 12 weeks). Furthermore, as another sensitivity analysis, we compared survival after the surgery without exclusion of the 567 patients who had recurrence or died within 6 months after surgery. We then compared the early and late groups using the univariate Cox proportional hazards models for RFS and OS. Event rates were estimated using the Kaplan–Meier method for RFS and OS. Statistical significance was set at *p* < 0.05. Statistical analyses were performed using JMP 7.0 (SAS Institute, Cary, NC) and R (R Foundation for Statistical Computing, version 4.1.2, Vienna, Austria).

**Table 1:** Background factors of the 3361 included PDAC patients

	Original cohort				IPTW cohort		
	Standard (*n* = 2681)	Delayed (*n* = 680)	*P* value	SMD	Standard (*n* = 3276)	Delayed (*n* = 3270)	SMD
Age (years)	69 (63–74)	70 (64–75)	0.001	0.127	69 (64–74)	69 (63–75)	0.004
Male Sex	1549 (58%)	374 (55%)	0.191	0.056	1875 (57%)	1914 (59%)	0.026
ECOG PS > 1	67 (2.5%)	12 (1.8%)	0.259	0.051	77 (2.3%)	69 (2.1%)	0.015
NAT +	537 (20%)	168 (25%)	0.007	0.112	688 (21%)	678 (21%)	0.007
Resectability			< 0.001	0.173			0.012
R	2226 (83%)	518 (76%)			2674 (82%)	2655 (81)	
BR	369 (14%)	135 (20%)			495 (15%)	503 (15%)	
UR	85 (3.2%)	27 (4.0%)			106 (3.2%)	111 (3.4%)	
CA19-9 level (U/ml)	82 (21–316)	80 (22–311)	0.688	-0.024	83 (21–318)	81 (22–310)	-0.001
Surgical procedure			< 0.001	0.171			0.021
PD	1610 (60%)	448 (66%)			2013 (61%)	2042 (62%)	
DP	999 (37%)	203 (30%)			1167 (36%)	1133 (35%)	
TP	72 (2.7%)	29 (4.3%)			96 (2.9%)	94 (2.9%)	
Operation time (min)	407 (296–511)	440 (343–541)	< 0.001	0.251	418 (305–518)	420 (321–516)	0.020
Intraoperative blood loss (mL)	494 (254–851)	568 (298–1010)	< 0.001	0.127	500 (260–884)	510 (250–919)	0.019
Postoperative complication	395 (15%)	215 (32%)	< 0.001	0.409	592 (18%)	590 (18%)	0.001
Pathological T factor			0.030	0.121			0.033
1	598 (22%)	134 (20%)			711 (22%)	693 (21%)	
2	1194 (45%)	318 (47%)			1481 (45%)	1452 (44%)	
3	837 (31%)	204 (30%)			1013 (31%)	1059 (32%)	
4	51 (1.9%)	24 (3.5%)			71 (2.2%)	66 (2.0%)	
Pathological N factor			0.300	0.066			0.006
0	1126 (42%)	308 (45%)			1385 (42%)	1385 (42%)	
1	1130 (42%)	270 (40%)			1374 (42%)	1376 (42%)	
2	425 (16%)	102 (15%)			516 (16%)	508 (16%)	
Pathological M factor, 1	36 (1.3%)	10 (1.5%)	0.798	0.011	43 (1.3%)	52 (1.6%)	0.023
Histology			0.786	0.044			0.025
Well	212 (7.9%)	52 (7.6%)			259 (7.9%)	241 (7.4%)	
Moderately	1585 (59%)	392 (58%)			1948 (59%)	1979 (61%)	
Poorly	758 (28%)	200 (29%)			915 (28%)	901 (28%)	
Other	123 (4.6%)	36 (5.3%)			153 (4.7%)	149 (4.5%)	
Residual tumor status R1	306 (11%)	84 (12%)	0.499	0.029	384 (12%)	398 (12%)	0.014

Continuous values are median (range), and categorical values are number of patients (%)

*BR* borderline resectable, *CA19-9* carbohydrate antigen 19–9, *DP* distal pancreatectomy, *ECOG PS* Eastern Cooperative Oncology Group Performance Status, *IPTW* inverse-probability-of-treatment weighting, *NAT* neoadjuvant therapy, *PD* pancreaticoduodenectomy, *PDAC* pancreatic ductal adenocarcinoma, *R* resectable, *SMD* standardized mean differences, *TP* total pancreatectomy, *UR* un-resectable

## Results

### Initiation time of S-1 adjuvant chemotherapy

**Fig. 2:**
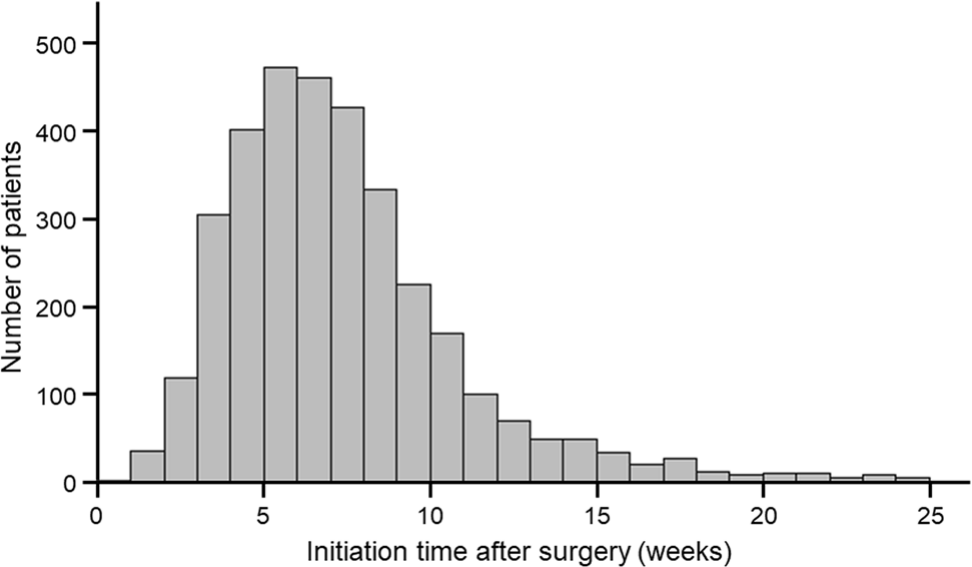
Distribution of initiation time for S-1 adjuvant chemotherapy. The bars indicate the number of patients based on the initiation time of S-1 adjuvant chemotherapy in the 3361 resected PDAC cases. *PDAC* pancreatic ductal adenocarcinoma

The distribution of initiation times for S-1 adjuvant chemotherapy among the included 3361 patients is shown in Fig. 2. The median time was 50 days (interquartile range: 38–66). Based on the distribution, the included 3361 patients were divided into two groups: 2681 (79.8%) initiating S-1 adjuvant chemotherapy within 10 weeks after the surgery (standard group), and 680 (20.2%) doing so beyond 10 weeks after the surgery (delayed group) (Fig. 1).

### Survival in the standard versus delayed groups

Before evaluating postoperative survival between the standard and delayed groups, we compared them in terms of preoperative, intraoperative, and postoperative factors. Table 1 shows the data for each group, with age significantly greater in the delayed group (*p* = 0.001). The percentage of borderline resectable and un-resectable PDAC also was significantly higher in the delayed group (*p* < 0.001), as was the percentage having had neoadjuvant therapy (NAT) (*p* = 0.007). Sex distribution, ECOG PS, and carbohydrate antigen 19–9 (CA19-9) level were not significantly different between the two groups. Regarding surgical factors, in addition to a greater proportion in the delayed group having pancreaticoduodenectomy (*p* < 0.001), operation time was longer (*p* < 0.001) and intraoperative blood loss greater in the delayed group (*p* < 0.001). Postoperative complications developed significantly more frequently with timing delay versus standard timing (*p* < 0.001). Regarding histological findings, PDAC was significantly more advanced with respect to T factor in the delayed group than in the standard group (*p* = 0.030), whereas N factor, histological type, and the incidence of R1 residual tumor status did not differ significantly between the two groups.

**Fig. 3:**
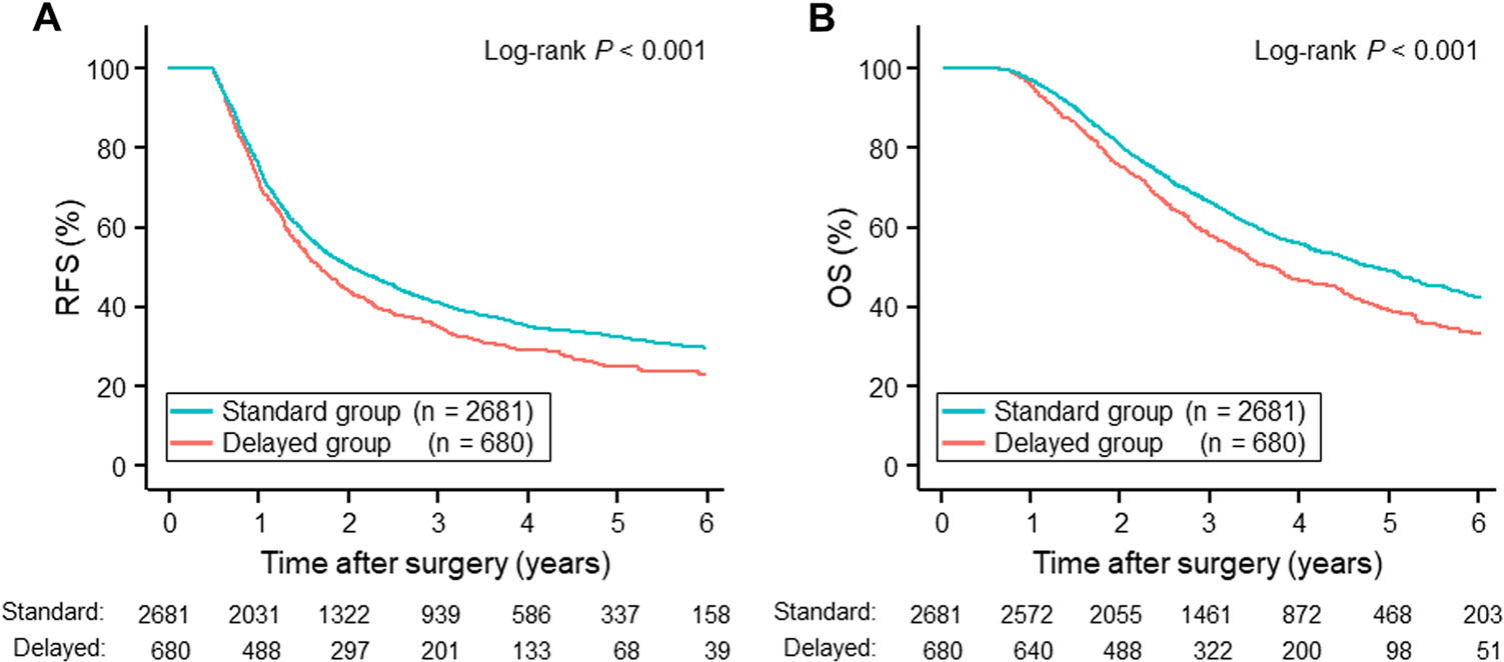
Survival curves after surgery. The curves indicate RFS (**A**) and OS (**B**) after surgery in the standard group (blue) and the delayed group (red). RFS and OS rates in the standard group were significantly better than in the delayed group (RFS: HR = 0.84, 95% CI = 0.76–0.93, *p* < 0.001; OS: HR = 0.77, 95% CI = 0.69–0.87, *p* < 0.001). The RFS MSTs in the standard and delayed groups were 24.2 months and 20.0 months, respectively; the OS MSTs in the standard and delayed groups were 57.0 months and 43.8 months, respectively. *P* values and number of patients at risk are shown in the panels. *CI* confidence interval, *HR* hazard ratio, *MST* median survival time, *OS* overall survival, *PDAC* pancreatic ductal adenocarcinoma, *RFS* recurrence-free survival

Given the background results, we compared postoperative survival between the two groups (Fig. 3). The 1-, 3-, and 5-year RFS rates after surgery in the standard group (76.1%, 40.8%, and 32.3%) were significantly higher than in the delayed group (72.2%, 34.6%, and 25.0%) (HR = 0.84, 95% CI = 0.76–0.93, *p* < 0.001), with median survival times (MSTs) of 24.2 months and 20.0 months, respectively. The 1-, 3-, and 5-year OS rates also differed between the two groups (standard group: 96.7%, 65.9%, and 48.7%; delayed group: 95.4%, 57.5%, and 38.7%; HR = 0.77, 95% CI = 0.69–0.87, *p* < 0.001), with MSTs of 57.0 months and 43.8 months, respectively.

We further compared the site of first recurrence between the two groups. In the standard group, the site was locoregional in 481 patients (28.9%), distant in 1025 (61.5%), and both in 160 (9.6%). In the delayed group, the site was locoregional in 133 patients (28.9%), distant in 278 (60.4%), and both in 49 (10.7%). The two groups did not differ significantly in this distribution (*p* = 0.789).

### Adjustment with IPTW analysis

**Fig. 4:**
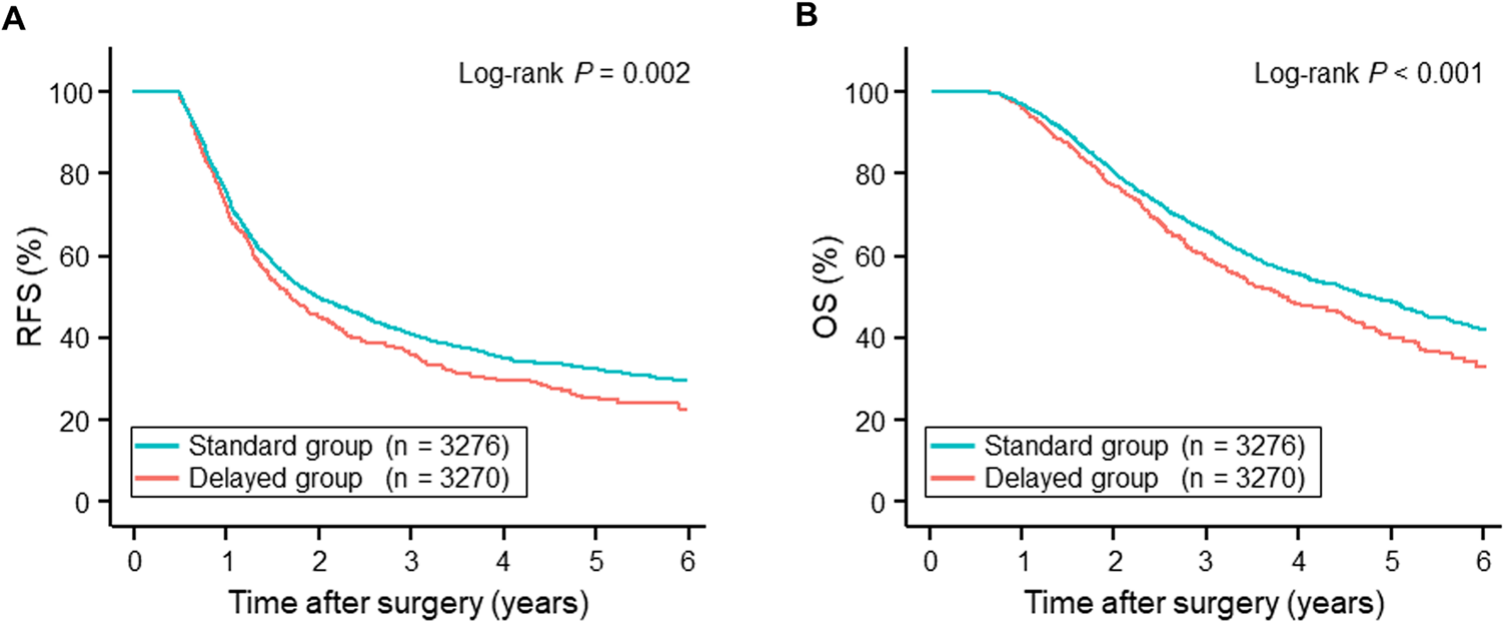
Survival curves after adjustment with IPTW analysis. The curves indicate RFS (**A**) and OS (**B**) after surgery in the standard group (blue) and the delayed group (red) after adjustment in the IPTW analysis. The RFS and OS rates in the standard group were significantly better than in the delayed group (RFS: HR = 0.86, 95% CI = 0.77–0.96, *p* < 0.001; OS: HR = 0.81, 95% CI = 0.71–0.92, *p* < 0.001). The RFS MSTs in the standard and delayed groups were 23.8 months and 20.4 months, respectively; the OS MSTs in the standard and delayed groups were 55.9 months and 45.2 months, respectively. *P* values are shown in the panels. *CI* confidence interval, *HR* hazard ratio, *IPTW* inverse-probability-of-treatment weighting, *MST* median survival time, *OS* overall survival, *PDAC* pancreatic ductal adenocarcinoma, *RFS* recurrence-free survival

The results of these comparisons suggested significant differences in OS and RFS between the standard and delayed groups, but the differences in some patient factors called for adjustment with IPTW analysis to verify the findings. After adjustment for the clinically relevant background factors in the IPTW analysis, no significant differences emerged in the background factors between the two groups (Table 1). We then compared RFS and OS between the groups and found that the differences remained significant (Fig. 4). The 1-, 3-, and 5-year RFS rates after the surgery in the standard group (respectively 75.9%, 40.7%, and 32.1%) were significantly higher than in the delayed group (72.6%, 35.6%, and 25.3%; HR = 0.86, 95% CI = 0.77–0.96, *p* < 0.001), with MSTs of 23.8 months and 20.4 months, respectively. The 1-, 3-, and 5-year OS rates also differed between the two groups (standard group: 96.6%, 65.5%, and 48.3%; delayed group: 95.6%, 58.9%, and 39.8%; HR = 0.81, 95% CI = 0.71–0.92, *p* < 0.001), with MSTs of 55.9 months and 45.2 months, respectively.

### Subgroup analysis

**Fig. 5:**
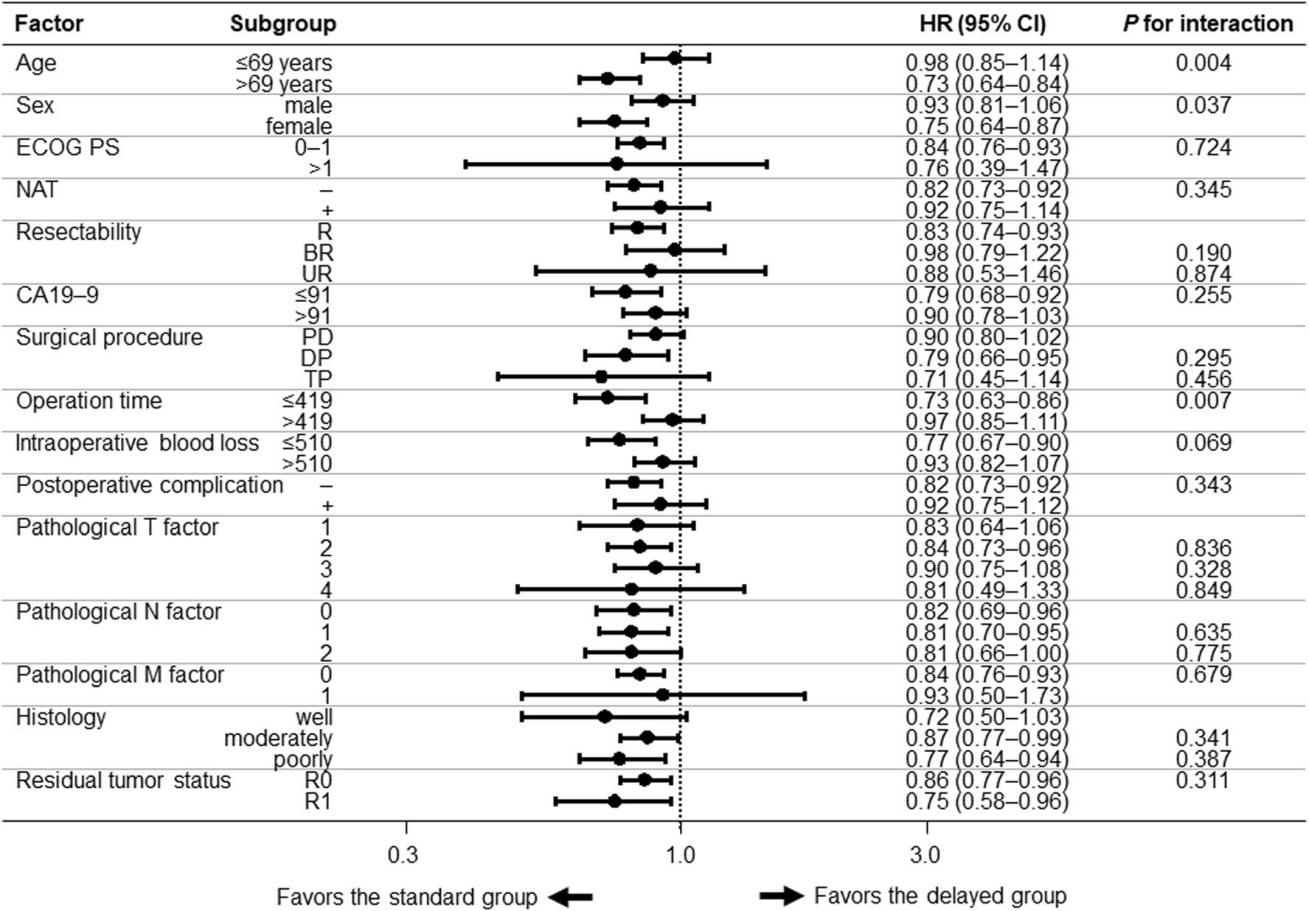
Forest plot of subgroup analysis of RFS. A black circle and horizontal line for each subgroup represent HR and 95% CI, respectively. *BR* borderline resectable, *CA19-9* carbohydrate antigen 19–9, *CI* confidence interval, *DP* distal pancreatectomy, *ECOG PS* Eastern Cooperative Oncology Group Performance Status, *HR* hazard ratio, *NAT* neoadjuvant therapy, *PD* pancreaticoduodenectomy, *R* resectable, *RFS* recurrence-free survival, *TP* total pancreatectomy, *UR* un-resectable

**Fig. 6:**
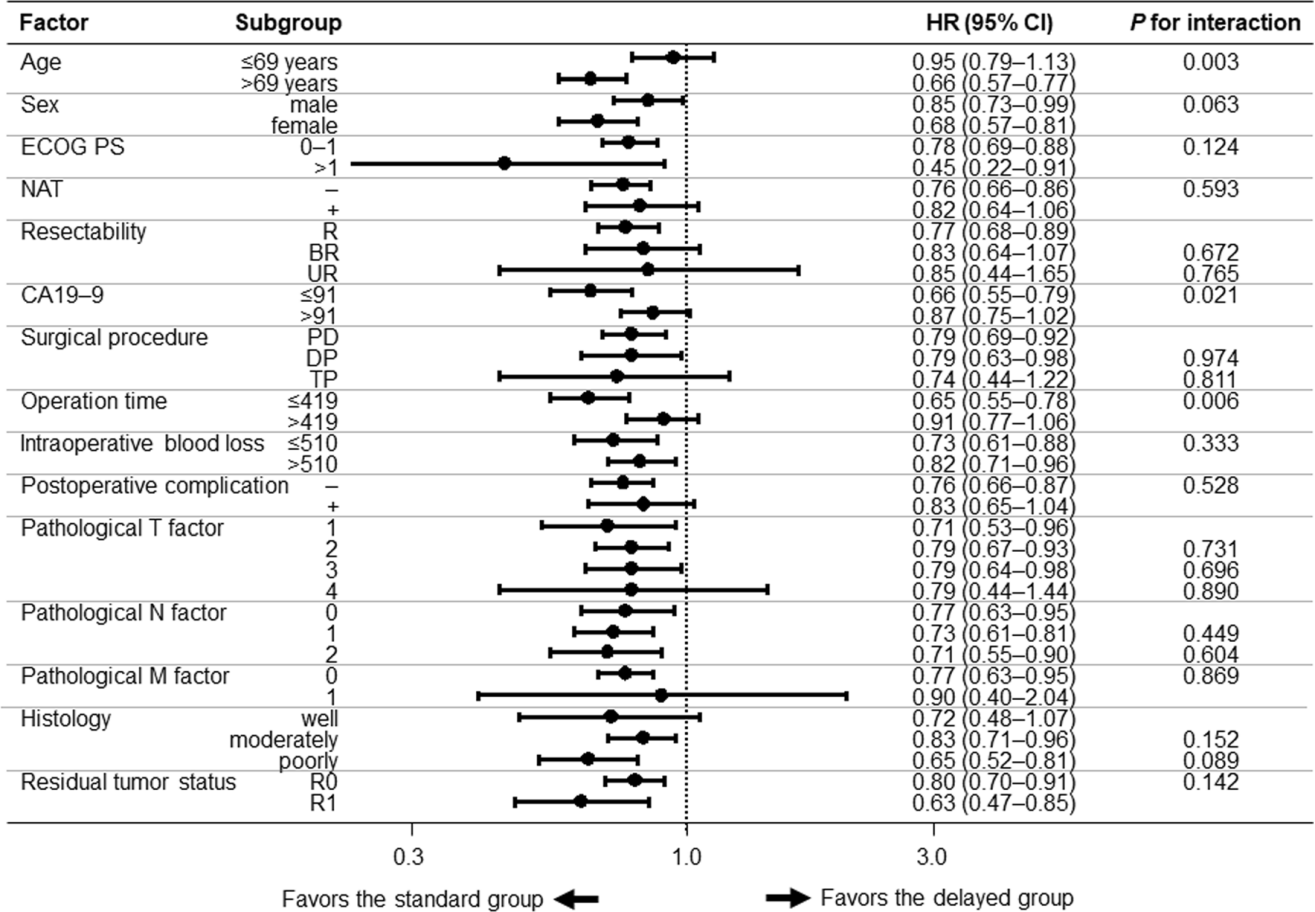
Forest plot of subgroup analysis of OS. A black circle and horizontal line for each subgroup represent HR and 95% CI, respectively. *BR* borderline resectable, *CA19-9* carbohydrate antigen 19–9, *CI* confidence interval, *DP* distal pancreatectomy, *ECOG PS* Eastern Cooperative Oncology Group Performance Status, *HR* hazard ratio, *NAT* neoadjuvant therapy, *OS* overall survival, *PD* pancreaticoduodenectomy, *R* resectable, *TP* total pancreatectomy, *UR* un-resectable

We performed subgroup analyses for RFS and OS to identify groups that experienced a survival effect from initiation of S-1 adjuvant chemotherapy within 10 weeks after surgery. Forest plots for the subgroup analyses for RFS and OS are shown in Figs. 5 and 6, respectively. In all subgroups, the standard group had superior RFS (HR = 0.71–0.98). There were significant additive interaction effects by age group (*p* for interaction: 0.004), sex (*p* for interaction: 0.037), and operation time (*p* for interaction: 0.007). Similarly, the standard group had superior OS (HR = 0.45–0.95) in all subgroups. There were significant additive interaction effects by age (*p* for interaction: 0.003), CA 19–9 (*p* for interaction: 0.021), and operation time (*p* for interaction: 0.006).

### Sensitivity analysis

Next, these results were verified in sensitivity analyses. With 8 weeks as the cut-off value, the RFS rate did not differ significantly between the two groups (log-rank *p* = 0.708), but OS was significantly better in the standard group (log-rank *p* = 0.0495). Using 12 weeks as the cut-off gave significantly better RFS and OS in the standard group (RFS: log-rank *p* = 0.006; OS: log-rank *p* < 0.001) (Supplementary Figure S1). Furthermore, in our sensitivity analysis of survival after surgery without exclusion of patients with recurrence or death within 6 months, RFS MSTs in the standard and delayed groups were 18.1 months and 17.3 months, respectively, and OS MSTs in the standard and delayed groups were 44.6 months and 38.5 months, respectively. (Supplementary Figure S2). These results verify the trend in survival differences between the standard and delayed groups.

### Comparison among four groups based on initiation timing

Finally, we compared RFS and OS among multiple groups stratified by initiation timing of S-1 adjuvant chemotherapy: ≤ 6 weeks, 6–10 weeks, 10–14 weeks, and > 14 weeks. The comparisons showed that although RFS and OS rates did not differ significantly between initiation at ≤ 6 weeks versus 6–10 weeks (RFS: log-rank *p* = 0.871; OS: log-rank *p* = 0.501), survival rates with initiation at ≤ 6 weeks were significantly higher than with initiation at 10–14 weeks (RFS: log-rank *p* = 0.058; OS: log-rank *p* = 0.023) or at > 14 weeks (RFS: log-rank *p* = 0.027; OS: log-rank *p* < 0.001) (Supplementary Figure S3).

## Discussion

This study was designed to investigate the survival impact of initiation timing for S-1 adjuvant chemotherapy, comparing 10 weeks after surgery with more than 10 weeks, based on real-world data from a nationwide survey. The results showed that survival was significantly better when the therapy was begun within 10 weeks after the surgery (the standard group) compared with later than 10 weeks after the surgery (the delayed group). The two groups differed in some respects, so we sought to confirm this significant difference using adjustment with an IPTW analysis. The results of the adjusted analysis confirmed the significant differences. These findings suggest a survival impact of beginning S-1 adjuvant chemotherapy within 10 weeks after surgery compared with later than 10 weeks in patients with resected PDAC.

So far, several studies have reported an association of the timing of adjuvant chemotherapy initiation and prognosis in resected PDAC patients. In an analysis of a multi-institutional national database registry including 5453 stage I and II PDAC patients with surgery followed by adjuvant therapy [14], Ma et al. found that adjuvant therapy administered within 28 to 59 days was linked to improved survival compared with earlier or later initiation. Despite the study’s size, a limitation was missing data on performance status, resectability status, presence of NAT, and surgical factors, such as operation time and intraoperative bleeding, and adjuvant therapy regimen, which can vary and is potentially associated with prognosis. Especially when considering initiation timing, information about the specific regimen is important. Other relevant studies also have a similar weakness involving varying chemotherapy regimens treated collectively in analyses [15, 16]. Several studies, however, have focused on initiation of adjuvant chemotherapy timed for specific regimens. For example, Murakami et al. reported improved survival when adjuvant chemotherapy with gemcitabine and S-1 was initiated within 20 days of PDAC resection compared with later initiation [26]. Valle et al. reported no significant difference in outcomes when the initiation of chemotherapy with 5-FU/folinic acid or gemcitabine was delayed until 12 weeks of resection, based on the European Study Group for Pancreatic Cancer–3 trial database [27]. In contrast, to the best of our knowledge, only one study has specifically addressed initiation timing for S-1 adjuvant chemotherapy in resected PDAC patients [17]. The results suggested an association of initiation > 51 days from surgery with inferior OS. Although the cut-off value of the initiation time was different from ours, the result is similar. However, that study included only 310 patients from three institutions, which the authors noted is a relatively small study cohort. Taking these previous studies together, the optimal initiation timing for S-1 adjuvant chemotherapy remained an open question, which led us to perform this secondary analysis of data from a large cohort of patients. Compared with previous studies, our investigation included a greater number of patients who received S-1 adjuvant chemotherapy at multiple institutions; however, the design was retrospective.

Based on our findings, we consider that postponement of S-1 adjuvant chemotherapy initiation beyond 10 weeks after surgery should be limited to situations in which it is unavoidable or indicated. From the surgical perspective, a recent study has shown that postoperative infectious complications may worsen prognosis by preventing timely adjuvant therapy in PDAC patients [28]. Therefore, considerable care should be taken to prevent delays related to postoperative complications and the time required to resolve them, which can force a postponement. In addition, some patient-related factors may be involved in delay of adjuvant therapy, such as inflammatory or nutritional status. In this context, when we consider the timing of S-1 adjuvant chemotherapy initiation, obstacles to early initiation are not uncommon in clinical practice, which in turn implies the importance of balancing initiation time with a good understanding of the patient’s condition. Thus, our results not only are a novel contribution regarding the impact of S-1 adjuvant chemotherapy initiation at a cut-off value of 10 weeks but also raise the clinical question of whether starting as soon as possible within that time frame is associated with a better prognosis. The question is an important one considering that the role of adjuvant chemotherapy is to eradicate residual cancer cells that could otherwise proliferate with support from growth factors and angiogenic factors related to surgical stress. The results of our four-group comparison showed no significant difference in RFS or OS between initiation in ≤ 6 weeks and 6–10 weeks, suggesting that early initiation within an optimal time frame offers limited benefit. Our results are not conclusive, however, and the question should be addressed in further rigorous studies.

Although this real-world data analysis involved a large cohort of PDAC patients, it has several inevitable limitations associated with its retrospective design. First, we applied statistical adjustment to minimize bias in the patient data, but some differences may have been retained between patients who could and could not initiate S-1 adjuvant chemotherapy quickly after surgery. For example, preoperative nutritional factors, co-morbidities, and frailty status, which are potentially associated with cancer prognosis, were not included in the database used in this study. Moreover, factors potentially reflecting the general postoperative condition of patients at S-1 administration were not included. Second, detailed information about the S-1 administration regimens is lacking, such as total dose, dose intensity, timing of dose reduction, rest period information, adverse effects, and how initiation time was determined in each patient. Especially, criteria for initiation of S-1 administration are not standardized across institutions and likely varied among them. Considering these limitations, despite the large number of PDAC patients included in this study, the evidence level cannot be considered high. Overcoming these limitations requires prospective studies comparing PDAC prognosis between two groups defined as having early or late initiation of S-1 adjuvant chemotherapy.

In summary, we analyzed data from a previous study to investigate the survival impact of initiating S-1 adjuvant chemotherapy within 10 weeks after surgery compared with starting it after 10 weeks. The results showed that with initiation within 10 weeks, survival was significantly better than with a later initiation. Although further studies are needed to prospectively validate the findings, the results offer some evidence for considering optimal timing for initiating S-1 adjuvant chemotherapy.

## Supplementary Information

Below is the link to the electronic supplementary material.

Supplementary file 1 (TIF 954 KB)

Supplementary file 2 (TIF 625 KB)

Supplementary file 3 (TIF 931 KB)

Supplementary file 4 (DOCX 51 KB)
