# Supplementary figures and images for: Standard versus delayed initiation of S-1 adjuvant chemotherapy after surgery for pancreatic cancer: a secondary analysis of a nationwide cohort by the Japan Pancreas Society
Source: J Gastroenterol. 2023 Jun 17;58(8):790–9. doi: 10.1007/s00535-023-01988-7 (PMC10366324; doi:10.1007/s00535-023-01988-7)

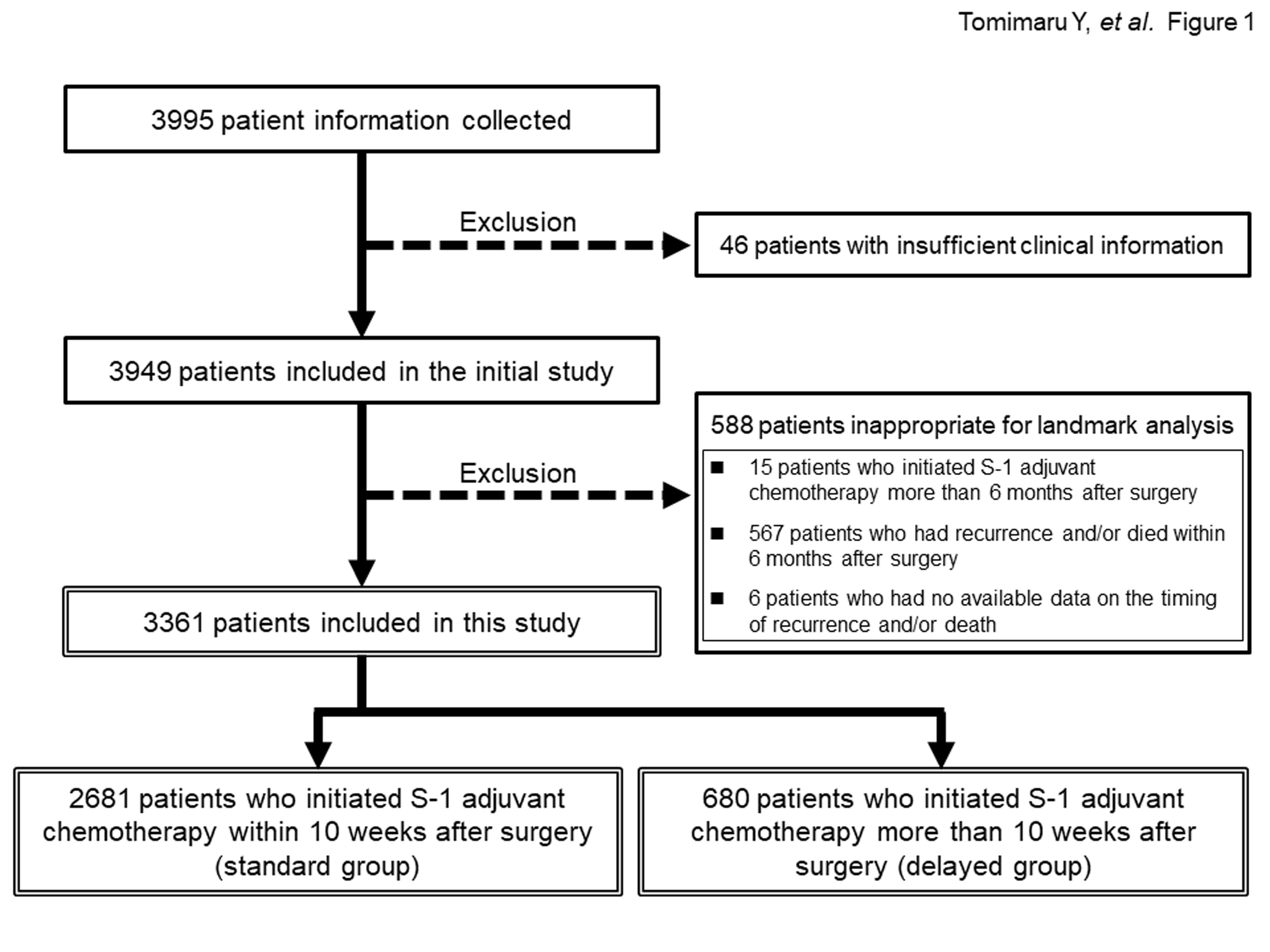

Supplement: Supplementary file 1 — (TIF 954 KB) [file 535_2023_1988_MOESM1_ESM.tif]

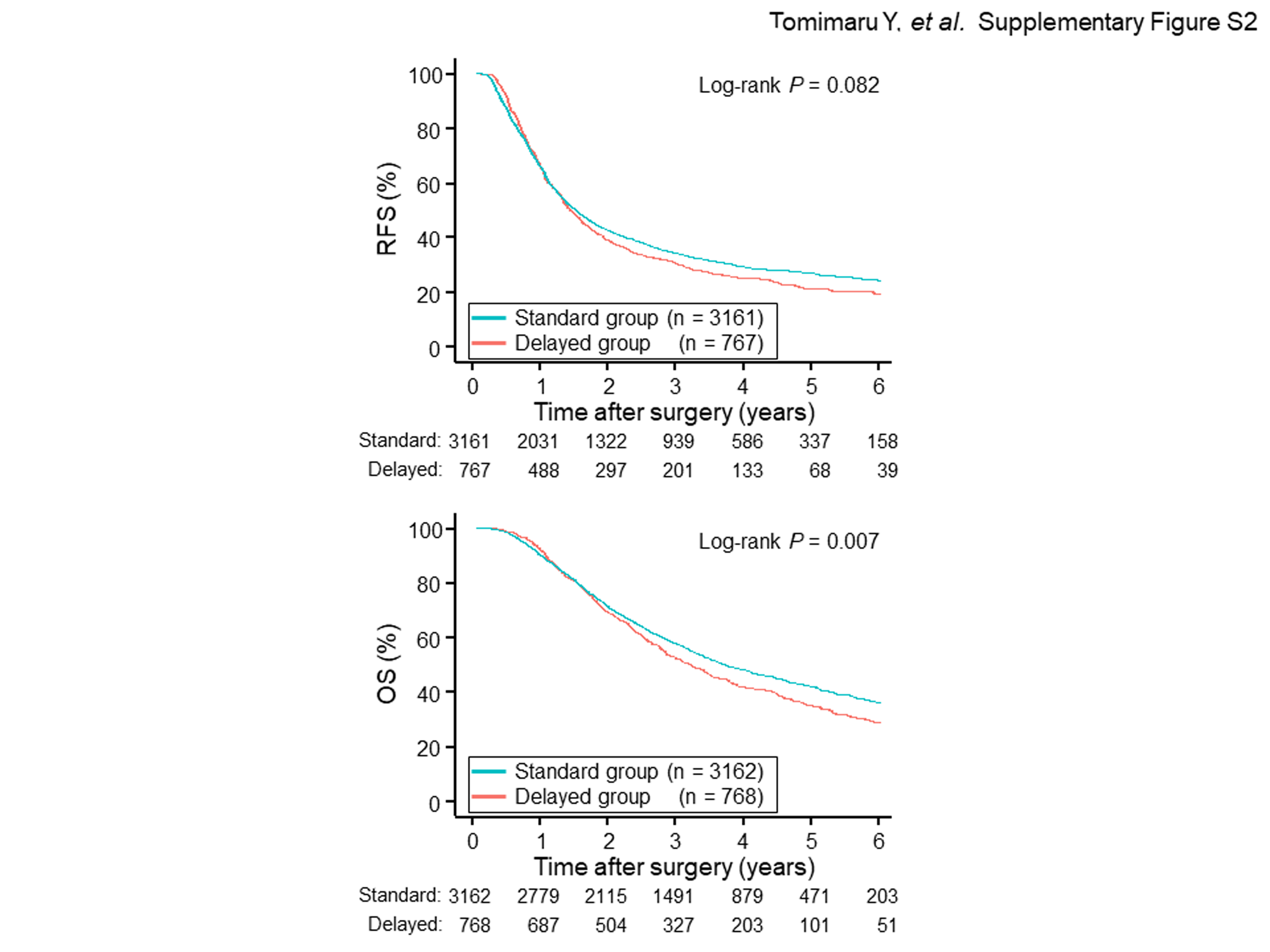

Supplement: Supplementary file 2 — (TIF 625 KB) [file 535_2023_1988_MOESM2_ESM.tif]

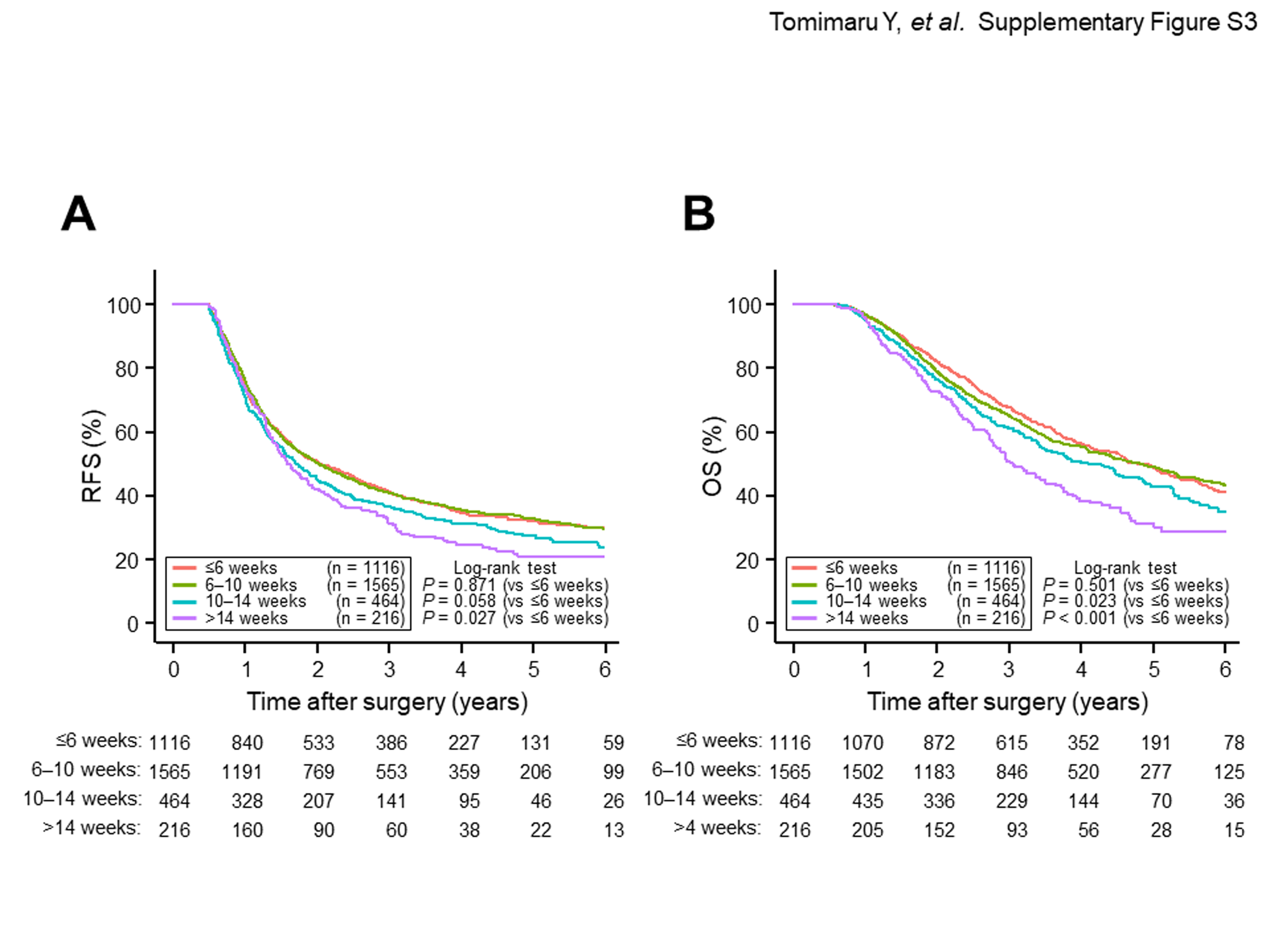

Supplement: Supplementary file 3 — (TIF 931 KB) [file 535_2023_1988_MOESM3_ESM.tif]
