# Supplementary material for: Standard versus delayed initiation of S-1 adjuvant chemotherapy after surgery for pancreatic cancer: a secondary analysis of a nationwide cohort by the Japan Pancreas Society
Source: J Gastroenterol. 2023 Jun 17;58(8):790–9. doi: 10.1007/s00535-023-01988-7 (PMC10366324; doi:10.1007/s00535-023-01988-7)
Supplement: Supplementary file 4 — (DOCX 51 KB) [file 535_2023_1988_MOESM4_ESM.docx]

**Supplementary Table S1.** List of individuals and institutions providing clinical information

| Individuals | Institutions |
| --- | --- |
| Yosuke Inoue | Cancer Institute Hospital, Japanese Foundation for Cancer Research |
| Yuichi Nagakawa | Tokyo Medical University Hospital |
| Akihiro Ohba | National Cancer Center Hospital |
| Hideki Takami | Nagoya University Hospital |
| Michiaki Unno | Tohoku University Hospital |
| Tomohisa Yamamoto | Kansai Medical University Hospital |
| Shoji Kawakatsu | Aichi Cancer Center |
| Tsuyoshi Hayashi | Teine Keijinkai Hospital |
| Hirohisa Kitagawa | Kurashiki Central Hospital |
| Ryota Higuchi | Tokyo Women's Medical University Hospital |
| Kenichi Hakamada | Hirosaki University Hospital |
| Minoru Kitago | Keio University Hospital |
| Yoshifumi Takeyama | Kindai University Hospital |
| Tatsuya Nomura | Niigata Cancer Center Hospital |
| Hisashi Ikoma | University Hospital, Kyoto Prefectural University of Medicine |
| Yasuhiro Fujino | Hyogo Cancer Center |
| Yuji Fujita | NTT Medical Center Tokyo |
| Hidetoshi Eguchi | Osaka University Hospital |
| Tomoyuki Yokota | Matsuyama Red Cross Hospital |
| Keiichi Kubota | Dokkyo Medical University |
| Yoshiki Hirooka | Fujita Health University Hospital |
| Yutaka Takeda | Kansai Rosai Hospital |
| Nakao Shirahata | Yamagata Prefectural Central Hospital |
| Masayo Motoya | Sapporo Medical University Hospital |
| Akira Matsushita | Nippon Medical School Hospital |
| Keiji Hanada | JA Onomichi General Hospital |
| Shugo Mizuno | Mie University Hospital |
| Ryo Takagi | Urasoe General Hospital |
| Masayuki Ohta | Oita University Hospital |
| Toshifumi Wakai | Niigata University Medical & Dental Hospital |
| Yusuke Ishida | Fukuoka University Hospital |
| Naoki Hiramatsu | Osaka Rosai Hospital |
| Yoichi Miyata | Asahi General Hospital |
| Yuichi Torisu | The Jikei University Hospital |
| Tsutomu Fujii | Toyama University Hospital |
| Masaji Tani | Shiga University of Medical Science Hospital |
| Shuji Suzuki | Tokyo Medical University Ibaraki Medical Center |
| Hiroaki Nagano | Yamaguchi University Hospital |
| Masahide Oshita | Osaka Police Hospital |
| Fuyuhiko Motoi | Yamagata University Hospital |
| Yoshinobu Okabe | Kurume University Hospital |
| Kei Yane | Tonan Hospital |
| Tsuyoshi Sano | Aichi Medical University Hospital |
| Ken Kawabe | National Hospital Organization Kyushu Medical Center |
| Atsushi Miyamoto | National Hospital Organization Osaka National Hospital |
| Naohiro Sata | Jichi Medical University Hospital |
| Shigeto Mizuno | Kindai University Nara Hospital |
| Takayoshi Nakajima | Meiwa Hospital |
| Hiromasa Ohira | Fukushima Medical University Hospital |
| Hideyuki Yoshitomi | Dokkyo Medical University Saitama Medical Center |
| Tsuyoshi Mukai | Gifu Municipal Hospital |
| Yoshinari Furukawa | Hiroshima Red Cross Hospital & Atomic-bomb Survivors Hospital |
| Takashi Hatori | International University of Health and Welfare, Mita Hospital |
| Joe Matsumoto | Obihiro Kosei Hospital |
| Makoto Shinzeki | Osaka Saiseikai Nakatsu Hospital |
| Fumihiro Okumura | Gifu Prefectural Tajimi Hospital |
| Yoichi Matsuo | Nagoya City University Hospital |
| Yoshifumi Morita | Hamamatsu University Hospital |
| Satoshi Tanno | IMS Sapporo Digestive Disease Center General Hospital |
| Takuro Yoshikawa | Saiseikai Suita Hospital |
| Akiko Takeshita | Okitama Public General Hospital |
| Tomoya Shamoto | Nagoya City University West Medical Center |
| Toru Niihara | Nanpuh Hospital |
| Toshifumi Ito | Japan Community Health care Organization Osaka Hospital |
| Shin Nakahira | Sakai City Medical Center |
| Naoki Hama | Ikeda City Hospital |
| Hiroyuki Hisai | Japanese Red Cross Date Hospital |
| Hiromichi Ishii | Matsushita Memorial Hospital |
| Hiroshi Yoshida | Iwaki City Medical Center |
| Toshifumi Matsumoto | Beppu Medical Center |
| Hiroyuki Sugimoto | Handa City Hospital |
| Yuki Ikeda | Steel Memorial Muroran Hospital |
| Akihiko Satoh | South Miyagi Medical Center |
| Go Murohisa | Seirei Hamamatsu General Hospital |
| Terumasa Yamada | Higashiosaka City Medical Center |
| Hidenori Takahashi | Osaka International Cancer Institute |
| Kenichi Sato | Tohoku Medical and Pharmaceutical University Hospital |
| Masafumi Naito | Suita Municipal Hospital |
| Noriyuki Kitagawa | Okaya City Hospital |
| Jiro Ohuchida | Miyazaki Prefectural Miyazaki Hospital |
| Shusaku Tsutsui | Itami City Hospital |
| Masahiro Tanemura | Rinku General Medical Center |

The institutions are ordered by number of patients.
